# Father's occupational exposure to carcinogenic agents and childhood acute leukemia: a new method to assess exposure (a case-control study)

**DOI:** 10.1186/1471-2407-8-7

**Published:** 2008-01-14

**Authors:** Maria Luisa Perez-Saldivar, Manuel Carlos Ortega-Alvarez, Arturo Fajardo-Gutierrez, Roberto Bernaldez-Rios, Maria de los Angeles del Campo-Martinez, Aurora Medina-Sanson, Miguel Angel Palomo-Colli, Rogelio Paredes-Aguilera, Armando Martínez-Avalos, Victor Hugo Borja-Aburto, Maria de Jesus Rodriguez-Rivera, Victor Manuel Vargas-Garcia, Jesus Zarco-Contreras, Janet Flores-Lujano, Juan Manuel Mejia-Arangure

**Affiliations:** 1 Epidemiologia Clínica, Hospital de Pediatria, Centro Medico Nacional "Siglo XXI", Instituto Mexicano del Seguro Social (IMSS), DF, Mexico; 2 Hematologia, Hospital de Pediatría, Centro Medico Nacional "Siglo XXI", IMSS, DF, Mexico; 3 Hematologia, Hospital General, Centro Medico Nacional "La Raza, IMSS, DF, Mexico; 4 Onco-Hematologia, Hospital Infantil de Mexico "Federico Gomez", SSa, DF, Mexico; 5 Hematologia, Instituto Nacional de Pediatria, SSa, DF, Mexico; 6 Oncología, Instituto Nacional de Pediatria, SSa, DF, Mexico; 7 Coordinacion de Salud en el Trabajo, Centro Medico Nacional "Siglo XXI", DF, Mexico; 8 Hospital General Regional No. 1 "Gabriel Mancera", IMSS, DF, Mexico

## Abstract

**Background:**

Medical research has not been able to establish whether a father's occupational exposures are associated with the development of acute leukemia (AL) in their offspring. The studies conducted have weaknesses that have generated a misclassification of such exposure. Occupations and exposures to substances associated with childhood cancer are not very frequently encountered in the general population; thus, the reported risks are both inconsistent and inaccurate. In this study, to assess exposure we used a new method, an exposure index, which took into consideration the industrial branch, specific position, use of protective equipment, substances at work, degree of contact with such substances, and time of exposure. This index allowed us to obtain a grade, which permitted the identification of individuals according to their level of exposure to known or potentially carcinogenic agents that are not necessarily specifically identified as risk factors for leukemia. The aim of this study was to determine the association between a father's occupational exposure to carcinogenic agents and the presence of AL in their offspring.

**Methods:**

From 1999 to 2000, a case-control study was performed with 193 children who reside in Mexico City and had been diagnosed with AL. The initial sample-size calculation was 150 children per group, assessed with an expected odds ratio (OR) of three and a minimum exposure frequency of 15.8%. These children were matched by age, sex, and institution with 193 pediatric surgical patients at secondary-care hospitals. A questionnaire was used to determine each child's background and the characteristics of the father's occupation(s). In order to determine the level of exposure to carcinogenic agents, a previously validated exposure index (occupational exposure index, OEI) was used. The consistency and validity of the index were assessed by a questionnaire comparison, the sensory recognition of the work area, and an expert's opinion.

**Results:**

The adjusted ORs and 95% confidence intervals (CI) were 1.69 (0.98, 2.92) during the preconception period; 1.98 (1.13, 3.45) during the index pregnancy; 2.11 (1.17, 3.78) during breastfeeding period; 2.17 (1.28, 3.66) after birth; and 2.06 (1.24, 3.42) for global exposure.

**Conclusion:**

This is the first study in which an OEI was used to assess a father's occupational exposure to carcinogenic agents as a risk factor for the development of childhood AL in his offspring. From our results, we conclude that children whose fathers have been exposed to a high level of carcinogenic agents seem to have a greater risk of developing acute leukemia. However, confounding factors cannot be disregarded due to an incomplete control for confounding.

## Background

Acute leukemias (AL) are the most frequent types of cancer in children under 15 years of age. The highest incidence rates in the world for AL have been reported for Latin American populations, and Mexico City is no exception. From 1996 to 2000, an average incidence rate of 58.4 cases per million children under 15 years of age has been reported for Mexico City [1]. Medical research has not established whether a father's occupational exposures are associated with the development of AL in his offspring. Pertinent studies had the following weaknesses [2-4]: 1) Information about occupational exposure was obtained from secondary sources or by using the occupation or the industrial branch as an indicator of the exposure; 2) the interviewed workers either had ignored the substances to which they were exposed or could not remember their past exposures; and 3) when exposure was characterized, only the duration of exposure was taken into account, with no consideration given either to the frequency or intensity of exposure, or to other variables such as the use of personal protective equipment. This has resulted in a misclassification of the exposure. Also, in these studies, when attempting to prove the occupational effect of a specific position or of exposure to a particular substance, the sample sizes have been unsatisfactory [2-4]. These are difficult problems to solve, because occupations and exposures to substances associated with childhood cancer are not very frequently found in the general population; therefore, the risks obtained have been inconsistent and inaccurate [5].

In this study, to assess exposure we used a new method, an index, which considered all parameters recommended to measure occupational exposure: industrial branch, specific position, use of protective equipment, substances at work, degree of contact with such substances, and time of exposure [2-4]. Even though there are only a few substances identified as having a potential leukemogenic effect, the underlying supposition to develop this new method to evaluate exposure was that it has not been possible to establish if such substances are related or not to the development of childhood leukemia because the frequency of exposure to each carcinogenic substance is very low. A method that grouped together each carcinogenic, or potentially carcinogenic, substance into an exposure index to carcinogenic substances was thought to solve the problem of the low frequency of exposure to each substance. Therefore, this exposure index was developed to allow us to obtain a grade that permitted the identification of individuals according to their level of exposure to known and to potential carcinogenic agents associated with an occupation, not necessarily specifically identified as a risk factor for leukemia [6]. The aim of this study was to assess the association between the level of father's occupational exposure to carcinogenic substances and the risk of his offspring in developing AL through the use of an occupational exposure index (OEI).

## Methods

From 1999 to 2000, a case-control study was performed with 193 children with AL and 193 controls consisting of children without AL, who were matched by age, sex, and institution of origin. All children resided in Mexico City and were under 16 years of age. The initial sample-size calculation was 150 children per group, assessed with an expected odds ratio (OR) of three and a minimum exposure frequency of 15.8%.

### Cases

In Mexico City, there are both public and private hospitals that treat children with AL. Private hospitals care for fewer than 5% of all children with cancer [7,8]. Out of the nine public hospitals that treat children with cancer and that were invited to participate in this study, in only four were we able to identify the population base in order to identify the controls. However, these four are the largest and most important hospitals in Mexico City and represent 88% of all the cases treated in public hospitals in Mexico City. All cases were diagnosed through cytochemical analysis of bone morrow aspirates; specific stains were used to differentiate acute lymphoblastic leukemia (ALL) from acute myeloblastic leukemia (AML). During that period, there was a total of 230 cases, 25 of which were excluded by the hospital in which they were diagnosed and 12 were also excluded because there was no information about the father (six single mothers, four abandoned mothers, one divorcee and one widow).

### Controls

We decided that controls should be selected from secondary-care hospitals that had referred children with AL to tertiary-care hospitals. Just as in other parts of the world, in Mexico, there are three levels of medical assistance: the primary-care group refers to treatment of patients by the family doctor, the secondary-care group is in charge of general medical specialties, and the tertiary-care group is responsible for giving treatment to difficult-to-manage diseases and for very specialized medical attention. The closer the level of medical attention is to the general public (*e.g*., primary care), the more the cases reflect the general population from which they arise. However, we chose children only from secondary-care hospitals for the following reason: Because a patient is assigned to a first-level clinic according to her/his address, there was a risk of over-matching due to father's occupation variable. This is because, in some cases, different companies have constructed apartment complexes into which their employees are crowded in the same community. In addition, we took into account the fact that including a hospitalized population would increase the participation rate of the controls; thus, we decided to include children coming from secondary-care hospitals as controls.

The control group was composed of children who had been admitted for short-stay surgery (hernioplasty, circumcision, orchidopexy), who lived with both their biological parents, and who could be matched with the cases by age (maximum 18-month difference) and gender. There were 415 children potentially eligible when these secondary-care hospitals were visited. However, the parents of 71 patients refused to participate, giving a no-response rate of 17%. It was not possible to locate the father of 46 patients. Out of the remaining 298 controls, only193 met the two criteria for pair matching by age and sex.

The protocol of the study was approved by the Ethics and Investigation Committee of the Instituto Mexicano del Seguro Social (No. 2003-243-003). The parents of each child signed an informed consent form.

### Data collection

Trained and standardized personnel conducted an individual, in-person interview with both parents of the indexed child. A questionnaire, adapted from the United States National Cancer Institute Questionnaire Modules [9], was used to obtain demographic information such as birth weight, gender, age of the father and of the mother during pregnancy, family history of cancer, and socioeconomic status. Each interview with the mother of a child with AL was conducted during the first two months after the diagnosis, and that of the father was completed within the first five months after the diagnosis. In the questionnaire, parents were asked to write what they thought was the reason their children had developed leukemia; in no case did they associate occupation as a cause.

The birth weight of the indexed child was divided into two groups, <3,500 g and ≥ 3,500 g. The parent's age was divided into two groups, >35 and ≤ 35 years of age. For both variables, the cut-off was determined as in other studies [10-12]. The level of crowding, part of a validated index in the Mexican population [13], was used as a proxy of the socioeconomic status. The crowding index is also that part of the socioeconomic level, which has been most frequently related to the risk of developing childhood leukemia [14]. The level of crowding, calculated as the number of people divided by the number of rooms in a home, was classified according to the criteria of Bronfman *et al*. [13]: not crowded, ≤ 3.5 persons per room; crowded, >3.5 persons per room. Parents were asked about cigarette smoking and alcohol consumption because, in a study carried out in Mexico City, it was determined that smoking and alcohol consumption by the parents are associated with the development of childhood AL [15] and that these variables are related to the occupation. The parents were also asked about their exposure to wood dust, fertilizers, pesticides, and hydrocarbons and derivatives thereof; such exposure was designated as "exposure to carcinogenic agents at home". All these factors were selected because, theoretically, they meet the confounding criteria described by Rothman and Greenland [16], who pointed out that a confounding factor must be a risk factor for the disease, must be associated with the exposure under study in the source population, and must not be in the causal pathway.

### Exposure assessment

#### Occupation

Through an individual, in-person interview, parents were asked to list all occupations in which they had been involved, for at least six months, during the following four periods: 1) the two years period prior to the conception of the indexed child; 2) during pregnancy; 3) during the breastfeeding period, and 4) after pregnancy either until diagnosis (for all cases) or until the date of the interview (for all controls). Each of the occupations was classified according to the International Standard Classification of Occupations version 1988 (ISCO-88) of the International Labour Organization [17].

#### Level of occupational exposure to carcinogenic agents

An exposure index (occupational exposure index (OEI)) was used in which the following indicators were considered for each position, with the information obtained from the labor history of the father: type of economic activity, type of specific position, use of personal protective equipment, toxic agents to which the individual was exposed, exposure frequency, exposure intensity, and degree of contact. Two specialists in occupational medicine were in charge of assigning to each reported occupation each of these indicators with a pre-established, weighted value, according to the probability of being in contact with carcinogenic agents in each occupation. The criteria were as follows:

##### a) Type of economic activity

According to the review by Savitz and Chen [2], two categories were considered, the first giving a value of 0 to the indicators not related to cancer in their offspring and the second, a value of 1 to those that were associated.

##### b) Type of specific position

A value was assigned according to the position occupied within the work, with a value of 1 given to office workers, 2 to supervisors, and 3 to those workers directly involved in the process.

##### c) Use of personal protective equipment

A value of 0 was given to those who used appropriate protective equipment, 1 to those who used inappropriate equipment, and 2 to those who did not use any equipment at all.

##### d) Exposure to carcinogens

The list suggested by the International Agency for Research on Cancer [18] was used. According to the evidenced degree of carcinogenicity, each group of compounds was weighted: A proved carcinogen group was assigned a value of 5; probable carcinogen in humans, a 4; possible carcinogen, a 3 and others, a 0. Substances of unknown composition were arbitrarily assigned a value of 1. Two databases were also used to identify and classify substances: the "Haz-Map Occupational Exposure to Hazardous Agents [19]," and the "Report on Carcinogens, 11^th ^Edition [20]."

##### e) Daily exposure frequency

This indicator was weighted with a value of 0.2 per hour on day(s) of exposure.

##### f) Exposure intensity or contact degree

A 1 value was given when there had been no contact with the substance; 2, if there had been contact by smell, but without handling the substance; 3, when the individual both smelled and handled the substance.

In order to calculate the OEI for each occupation, the values for industrial branch (*a*), type of position (*b*), and use of protective equipment (*c*), were added together; to this value, was added the summation of the product of the values for each substance (*d*), for the frequency of exposure (*e*), and for the degree of contact (*f*), giving the formula *OEI = a+b+c+Σdef*.

When applying the formula and in accordance with the validation, "high exposure" was considered to be ≥ 25 points and "non-high exposure" to be <25 points, where "non-high exposure" includes moderate, low, and null levels.

### Instrument validation

Workers (n = 52) from nine different industries were studied [6]. The companies were selected considering the industrial branch, their processes, and their raw materials; they were distributed according to the risk of exposure to low-, medium-, and high-risk carcinogenic agents, with three companies in each category. A work environment sensory recognition and the application of the assessment instrument were applied independently to workers from different areas to have representation of the different positions involved in the process. An exposure index evaluation was carried out to assess exposure to carcinogenic agents, considering the above-mentioned indicators; it was assessed with the highest probability value of exposure to carcinogenic agents. The consistency and validity of the index were assessed by a questionnaire comparison, the sensory recognition of the work area, and an expert's opinion. Although the responses to the questionnaire were not validated for every individual interviewed, a sensory analysis of the specific position was done in order to evaluate consistency with the response given by worker. The person who conducted the interview did not know the results of the sensory analysis and vice versa. When checking the questionnaire, the expert classified workers according to high, moderate, and low exposure on two occasions in a month interval and showed a high consistency in classifying them, with a weighted Kappa of 0.806. The sensory recognition report was also evaluated twice by the expert, with a weighted Kappa of 0.973. For this reason, the sensory recognition and its interpretation by an expert were chosen as a gold standard by which to measure the validity of the index obtained from the questionnaire. Receiver Operating Characteristic (ROC) curves were plotted to show the best cut-off level for the index. It was found that the exposure index did not differentiate between high and moderate degree of exposure, nor between moderate and low. Sensitivity and specificity, but especially likelihood ratio, were increased when both low- and moderate degree of exposure were combined. The cut-off level to distinguish between these degrees of exposures was 25 points, with a 100% sensitivity level, a 93% specificity, and a 16.66 likelihood ratio. A limiting factor that was observed was that, when considering the number of years of exposure, the specificity, and the likelihood ratio decreased. This situation did not affect this study, for the exposure time necessary for the child to develop AL seems to be not greater than two years [21].

### Statistical analyses

A simple, stratified, and logistic regression analysis was performed to calculate the OR with 95% CI. This analysis was performed for four life periods: Two years before the conception of the indexed child, during pregnancy, during breastfeeding, and the period after breastfeeding until either diagnosis (for the cases) or until the date of the interview (for the controls). Analyses were also performed 1) without including the breastfeeding period and 2) including one more period in which global exposure was analyzed over all four periods.

A complete model was built. It included 1) the father's occupational level of exposure (beta); 2) all the potentially confusing variables (family cancer history, sex of child, age of child at the time of diagnostic or interview; weight at birth, crowding level, father's and mother's age at pregnancy, father's and mother's alcohol consumption, father's and mother's tobacco use, and exposure to carcinogenic agents at home) (gammas); and 3) all the potential interactions between the father's occupational level of exposure and all the potentially confounding variables (deltas) [22].

By constructing a model in which all the interactions were eliminated and by comparing the -2 likelihood (-2LK) to the complete model, a value of P = 0.64 was obtained; therefore, it was concluded that the interactions did not have an influence. Then, the model with all the potentially confounding variables was compared to another model without these variables; from the result (P = 0.003), we concluded that there was confounding.

Those variables that had a difference lower than 10% between the crude OR and the adjusted OR were discarded. Several partial models were run until a P > 0.10 was obtained when comparing the -2LK of the complete model to the -2LK of the partial model.

## Results

### Population description

In this study, 193 cases and 193 controls were analyzed. There were 163 cases of ALL (84.5%) and the rest of the cases were myeloid leukemias. For the sociodemographic variables, groups were similar; however, the cases showed a greater frequency of being positive for the following variables: family history of cancer, father's cigarette smoking during child's gestation, mother's cigarette smoking during the breastfeeding period, and exposure to carcinogenic agents at home (Table 1).

**Table 1:** Association of different factors with acute leukemia in children from Mexico City

**Variables**	**Cases (N = 193)**		**Controls (N = 193)**		**Crude OR [95% CI]**
		
	**N**	**%**	**N**	**%**	
*Type of leukemia*					
*ALL*	163	84.5			
*AML*	30	15.5			
*Institution*					
*IMSS*	122	63.2	124	64.2	
*SSa*	71	36.8	69	35.8	
*Family history of cancer*	88	45.6	67	34.7	1.58 [1.05,2.38]
*Sex: Male*	110	57.0	115	59.6	0.90 [0.60,1.35]
*Age: 2–5 years old*	59	30.6	53	27.5	1.16 [0.75,1.81]
*Child's weight at birth: >3,500 g*	60	31.1	56	29.0	1.10 [0.71,1.71]
*High crowding*	80	41.5	71	36.8	1.22 [0.81,1.83]
*Paternal age during pregnancy: >35 years old*	26	13.5	24	12.4	1.10 [0.61,1.99]
*Maternal age during pregnancy: >35 years old*	13	6.7	16	8.3	0.80 [0.37,1.71]
*Paternal alcohol consumption before pregnancy*	172	89.1	172	89.1	1.00 [0.53,1.90]
*Maternal alcohol consumption before pregnancy*	139	72.0	154	79.8	0.65 [0.41,1.04]
*Paternal cigarette smoking before pregnancy*	118	61.1	105	54.4	1.32 [0.88,1.98]
*Paternal cigarette smoking during pregnancy*	107	55.4	88	45.6	1.49 [0.99,2.22]
*Paternal cigarette smoking after pregnancy*	117	60.6	110	57.0	1.16 [0.77,1.74]
*Maternal cigarette smoking before pregnancy*	35	18.1	49	25.4	0.65 [0.40,1.06]
*Maternal cigarette smoking during pregnancy*	7	3.6	10	5.2	0.69 [0.26,1.85]
*Maternal cigarette smoking during breastfeeding*	9	4.7	3	1.6	3.10 [0.83,11.62]
*Exposure to carcinogenic agents at home*	46	23.8	29	15.0	1.77 [1.06,2.96]

ALL, acute lymphoblastic leukemia; AML, acute myeloid leukemia; IMSS, Instituto Mexicano del Seguro Social; SSa, Secretaria de Salud; OR, odds ratio; CI, confidence interval

### Occupation

Table 2 shows the various occupations that each father had before conception of the indexed child and for which a non-significant increased risk of developing AL was reported. The only occupation that showed a statistically significant increased risk was insurance agent. Occupations that remained as risk occupations during the four periods were the following: insurance agent, farmer, machinery operator, mechanic, packer, and builder (data not shown).

**Table 2:** Analysis of the father's most frequent occupations, two years before conception of indexed child.

**Occupation**	**Cases**		**Controls**		
		
	**N**	**%**	**N**	**%**	**Crude OR [95% CI]**
*Accountant*	4	2.1	5	2.6	1.0 Reference
*Motor vehicle driver*	17	8.8	12	6.2	1.77 [0.39,8.00]
*Builder*	17	8.8	10	5.2	2.12 [0.46,9.81]
*Machinery operator*	14	7.3	7	3.6	2.50 [0.51,12.35]
*Insurance agent*	11	5.7	1	0.5	13.75 [1.20,156.65]
*Mechanic*	9	4.7	4	2.1	2.81 [0.48,16.43]
*Cleaning person*	8	4.1	7	3.6	1.42 [0.27,7.51]
*Farmer*	7	3.6	3	1.6	2.91 [0.44,19.23]
*Packer*	7	3.6	3	1.6	2.91 [0.44,19.23]
*Carpenter*	6	3.1	4	2.1	1.87 [0.30,11.63]

OR, odds ratio; CI, confidence interval

### Exposure level

For this variable, all occupations for each period were considered; a period was classified as "exposed" if the index indicated that the father had been "highly exposed" in at least one of his occupations during that period (Table 3). By using logistic regression, it was possible to conclude that interactions were not an influence, but that confounding did exist. The final logistic regression model included nine variables for the father's occupational exposure level: age, gender, institution where the child received treatment, maternal occupation, family history of cancer, weight at birth, socioeconomic status, paternal cigarette smoking, and exposure at home. The adjusted OR showed a significantly increased risk in all periods, with exception of the pregestational period that reported a non-significant increase in OR.

**Table 3:** Paternal level of occupational exposure to carcinogenic in the life of the indexed child.

**Variables**^a^	**Cases (N = 193)**		**Controls (N = 193)**		**Crude OR [95% CI]**	**Adjusted^b ^OR [95% CI]**
			
	**N**	**%**	**N**	**%**		
*At least one occupation with high exposure before conception of indexed child*	45	23.3	28	14.5	1.79 [1.06,3.02]	1.69 [0.98,2.92]
						
*At least one occupation with high exposure during pregnancy period of indexed child*	42	21.8	26	13.5	1.79 [1.05,3.06]	1.98 [1.13,3.45]
						
*At least one occupation with high exposure during breastfeeding period of indexed child*	39	20.2	22	11.4	1.97 [1.12,3.47]	2.11 [1.17,3.78]
						
*At least one occupation with high exposure after birth of indexed child*	54	28.0	29	15.0	2.20 [1.33,3.64]	2.17 [1.28,3.66]
						
*At least one occupation with high global exposure, considering all four periods*	62	32.1	35	18.1	2.14 [1.33,3.44]	2.06 [1.24,3.42]

OR, odds ratio; CI, confidence interval

^a^Only "highly exposed" father's values are reported; values taken as a reference and which correspond to the "non-highly exposed" fathers are not shown.

^b^This analysis was adjusted by age, sex, source institution, level of crowding, paternal cigarette smoking, exposures at home, and mother's occupation.

Because some fathers reported more than one occupation for the period after the birth of the indexed child, the number of occupations with high exposure to carcinogenic agents was analyzed. As shown in Table 4, the greater the number of occupations with a high exposure, the greater the risks that showed a significant trend (*p *< 0.001).

**Table 4:** Trend analysis on the number of occupations with high paternal exposure to carcinogenic agents after indexed child was born.

**Variables**	**Cases (N = 193)**		**Controls (N = 193)**		**Crude OR 95% CI**
		
	**N**	**%**	**N**	**%**	
*No occupation with high exposure*	139	72.0	164	85.0	1.0 Reference
					
*One occupation with high exposure*	43	23.8	28	14.5	1.81 [1.07,3.07]
					
*Two or more occupations with high exposure*	11	4.2	1	0.5	12.98 [1.65,101.78]

OR, odds ratio; CI, confidence interval

Chi square trend = 12.78 (p < 0.001)

## Discussion

This is the first study in which the father's occupation is assessed as a risk factor for the development of childhood AL in his offspring by using an OEI to carcinogenic agents.

From the first published study by Fabia and Thuy in 1974 [23], which showed the association between the father's occupation and the development of malignant diseases in his offspring, several articles have been published on this topic; however, these studies have been inaccurate and have had inconsistent results [2-4]. For this reason, Linet *et al*. in 2003 [24], when classifying the evidence for risk factors for AL into known, stimulating, and limited, classified parental occupational exposures as a risk factor of limited evidence.

One way to increase accuracy in this type of studies was recommended by Ward *et al. *[25]. They pointed out that it is better to conduct studies with large sample sizes when studying specific substances as risk factors, because these types of exposures are very rare among general population. The present study did not require a large sample size; the strategy used was to diminish the variability in measures by using a strict measuring protocol for the exposure trough the use of an exposure index [26].

Regarding the possibility of selection bias with the cases, it is important to point out that the children included in this study were drawn from highly specialized, public pediatric hospitals that, on the whole, give treatment to about 95% of the cases of childhood AL in Mexico City [7,8]. Although these hospitals had only 88% of all cases in public hospitals, these cases represented 100% of the cases for which it was possible to identify an appropriate control; that is, for which it was possible to identify the secondary-care hospital that had referred them to the tertiary-care hospital for leukemia diagnosis and treatment.

For the controls, individuals were included from general hospitals under the aegis of the two institutions from which the cases were obtained: Instituto Mexicano del Seguro Social (Social Security Mexican Institute) and Secretaria de Salud (Health Secretariat). The hospitals were located in different parts of Mexico City: south, north, center-west, and east sections of the city. Controls were not drawn from the same tertiary-care hospitals from which the cases were taken, because the diseases that these hospitals treat are associated with different risk factors that would make them totally different from the study's base population [26].

In this study, hospital controls obtained from medical assistance centers were used. Such centers work as reference units for the hospital from which the cases were drawn. If any of the controls were to have developed AL, the case would have gone directly to the case-source hospitals. Moreover, because of the lack of differences between sociodemographic variables among groups, we could conclude that cases, as well as controls, came from the same population base [27].

In regard to interviewer bias, cases and controls were interviewed under similar conditions; however, the cases reported greater frequencies for some non-occupational exposures. We could not eliminate the possibility that recall bias had been present; however, we applied techniques suggested to eliminate such bias: a structured and standardized questionnaire, which provided memory aids, was used; trained personnel obtained data as accurately as possible, and hospital controls were used [28,29]. The interviewer bias was limited, because the trained personnel acting as interviewer did not know the main hypothesis for the study and they were standardized.

The way to obtain the necessary information to estimate the index was through direct questioning, which is considered to increase the participation rate of both cases and controls; direct questioning is also considered to increase the reliability of the information so obtained [30]. There is no evidence available to suppose that fathers from either group would over-report the frequency of occupational exposures. It is possible that fathers could not remember all exposures throughout their work life; however, such lack of precision would be similar for parents from the cases and the controls; therefore, the estimated ORs would be an underestimation of the real OR [31]. It has been recommended that, in epidemiological studies, interviews with fathers of children with cancer be performed before they seek an explanation to their children's disease, because such situation could bias their answers [30]. In this study, we had the advantage that, for 100% of the cases, interviews with the mothers were performed within the first month after the diagnosis had been made and the father's interview within the first five months. Moreover, none of the fathers stated, in any of the questionnaires, that they thought that one of the causes for their children's illness could have been an occupation that they had had.

The prevalence of occupational exposure to carcinogenic agents was from 11.4 to 15.0% among the controls and from 20.2 to 28.0% among the cases. This frequency was high because this index grouped together all the known and potentially carcinogenic substances reported by the worker and not one substance in particular. At present, it is not possible to state that the frequency of exposure to carcinogenic agents in the studied population was greater than that in the rest of the population, due to the fact that no other study has used the instrument that we employed to evaluate exposure. Nonetheless, it is known that about 23% of the working population in the European Union is exposed to carcinogenic substances [32].

Confounding was controlled by a logistic regression analysis. A conditional regression analysis was not performed because none of the matching variables for the study was considered a risk factor for the disease, an implicit criterion for a variable to be considered as a true confounding factor; therefore, the matching variables in the analysis should be maintained [33]. In this study, through logistic regression analysis, we determined that the risks found were confounded by the occupation of the mother. Maternal occupation has been less studied than paternal occupation and associations have also been less consistent [2,4]. However, in two recent studies, an increase in risk of developing AL was identified for offspring of mothers who presented occupational exposures to electromagnetic fields [34] and solvents [35] during pregnancy. The cut-off levels used for the mother's age (≤ 35 and >35) and for the index of child's weight at birth (≤ 3,500 and >3,500 g) were those that are most frequently reported in medical literature [11,12]. There are no consistent data showing that the mother's age is a risk factor for her offspring to develop childhood leukemia; Little interpreted this inconsistency as the result of maternal age may be more a reflection of sociological, rather than biological, influence [10]. The effect of mother's age may reflect the increase in the frequency of non-disjunction during oogenesis, which increases with maternal age, and polygenic or imprinting mechanisms may involve a tendency to non-disjunction; these mechanisms may have implications for the etiology of leukemia in children [36]. The most frequently reported birth weight is >3,500 g [11]; in recent studies, the cut-off level of >4,000 g has been used, but a weight between 3,000 and 3,500 g is considered the average weight [37]. There have been no consistent data to show that this is a risk factor for childhood leukemia, however, a proposed mechanism by which it may be related to leukemia is that overweight at birth may be a result of high levels of growth factors in the uterus and that these growth factors may increase the risk to acute leukemia when inducing a proliferative stress in the bone marrow [37].

Another factor evaluated as a possible confounding variable was exposure to carcinogenic substances at home. We decided to include this factor because it has consistently been associated with acute leukemia [38,39]; the most studied cases of exposure have been occupational or residential exposure, exposure at home has been less studied [40]. More studies have been done on hydrocarbons associated with pesticides and here is where the strongest associations have been found [40,41]. The mechanism(s) by which some hydrocarbons, including those contained in pesticides, increase the risk to develop cancer is(are) not thoroughly understood [42]. Some mechanisms are chromosomal damage; disruption of cell division; and reduction in host resistance to cancer-initiating viruses, such as the Epstein-Barr virus, which can provoke a breakdown in the immune surveillance [42]. Some compounds in this group of chemicals are immunotoxic [43].

Using occupations and industrial branches as risk factors for the development of cancer in their offspring has given rather inaccurate results [44]; it is for this reason that most recent studies have focused on using occupation and economic activity to deduce the substances to which workers are exposed by using exposure matrixes [45]. Some studies that deduced exposures obtained information on occupation from secondary sources. On this point, Swaen *et al*. [46] have commented that it is possible, when information is obtained from cancer records or secondary sources, that there are false-positive results in studies on cancer and occupational exposures. Such false positives would be reduced when the information obtained permits the analysis of the relationship between doses and examine the phenomena. In this study we were able to estimate the trend, by finding an exposure gradient for the number of occupations with high exposure in the period after the birth of the indexed child and with statistically significant values in the trend assessment. In the present study, when the job position was evaluated in a specific way, an association was found with insurance agents; however, this finding could be a result of chance.

The use of experts is another strategy to assess exposure, which is not exempt from misclassification errors [47]. Reiner *et al. *[48], stated that exposure misclassification has been the main limitation in studies assessing parental occupational exposure as a risk factor for the development of diseases in offspring. This has given rise mainly to suggestions to improve the quality of questionnaires and of data-collection techniques [49].

Another proposal is the development of more sophisticated methods to assess exposure [24], preferably in a quantitative way [50], through the use of estimation models that incorporate the phenomena-determining factors (frequency, intensity, duration, etc); this would increase accuracy and reliability of the exposure estimation. Two articles were recently published on new instruments to assess occupational exposures for studies on childhood AL. One of them suggests the use of a questionnaire with specific work modules to achieve a better description of exposure [48]; the other study assesses exposure to pesticides, suggesting the use of icons to facilitate the worker's understanding [51]. These instruments were used to try to improve the measurement of exposure, but none of the instruments evaluated occupational exposure in a quantitative or semi-quantitative way.

The main strength and contribution of the present study is that, through use of the OEI, when obtaining information, we were able to take into account all these suggestions, integrate them into the study, and then use them in a formula to calculate a value that that represented the level of exposure.

Another strength of this study was the analysis of the father's exposures during different periods in the life of the indexed child. A cohort study, in which the father's occupational exposure to fungicide was assessed as a risk of developing cancer in his offspring, classified exposure as low, medium, or high [52]. That study identified risks to highly exposed in the periods 1) prior to conception, 2) during pregnancy, and 3) after the birth of indexed child; the ORs were 1.7, 1.3, and 1.7, respectively, but again with broad CIs and with P values in the trend test having no statistical significance. These findings coincide with the present study, in which farmers were found to be an occupation with high risks in all four periods. Moreover, those data coincide with the fact that the incidence of AL is higher in the southwestern part of Mexico City, where there are still agricultural zones [53]. In another study that used exposure windows, an association was found only for the father's exposure to plastic materials during period prior to conception [54]. McKinney *et al*., found risks only for exposure to exhaust fumes and inhaled particles of hydrocarbons during the period prior to conception, which was the only one evaluated [55].

A weak point of this study was the small size of the sample. Although most of the adjusted ORs were significant statistically, it is not possible to disregard the role of chance.

Another weak point is that neither the population mix nor exposure to infections was considered as possible confounding variables. There is sufficient evidence to think that childhood AL has an infectious etiology [56] and that fathers, laboring in certain occupations and having frequent contact with other people at the time of the child's birth, can be the source of contagion for the child [57]. Additionally, there is the possibility that the working population in search of a better job may need to migrate from rural to urban populations. This is the reason why, when considering studies of paternal occupation, evaluation of population migration has been recommended [56]. In the present study, this variable was not evaluated either; however, when assessing the number of children who had been born in a rural community and now live in Mexico City (urban community), we found that only seven cases and six controls had been born in a rural community (OR 1.17; 95% CI 0.38–3.5). Due to such a small number of individuals, it was not feasible to evaluate whether migration from a rural zone to an urban one differed, depending on the level of exposure of the father to carcinogenic substances and much less on a specific occupation of the father.

## Conclusion

With the results obtained from this study, we concluded that, among the children of fathers exposed to a high level of carcinogenic substances at work, there seemed to be a greater risk of developing AL. However, confounding factors cannot be disregarded due to incomplete control for confounding.

## Competing interests

The author(s) declare that they have no competing interests.

## Authors' contributions

MLPS participated in the design of the study and in the analysis of the data, wrote the first and last draft of the manuscript, and provided guidance to all aspects of this project. MCOA participated in the design of the study and in the analysis of the data and wrote the first draft of the manuscript. JMMA conceived of the study, participated in its design and in the analysis of the data, corrected the first draft and final manuscript, provided guidance to all aspects of this project, and obtained funding. AFG and VHBA participated in the design of the study and in the analysis of the data, and provided guidance in some aspects of this project. MLPS, RBR, MACM, AMS, MAPC, RPA, AMA, MJRR, VMVG, JFL, and JZC registered, recorded, and analyzed the data. All authors read and approved the final manuscript.

## Pre-publication history

The pre-publication history for this paper can be accessed here:


